# Supplementary material for: Distinct translatome changes in specific neural populations precede electroencephalographic changes in prion-infected mice
Source: PLoS Pathog. 2022 Aug 12;18(8):e1010747. doi: 10.1371/journal.ppat.1010747 (PMC9401167; doi:10.1371/journal.ppat.1010747)
Supplement: S1 Methods — Included are method details for 1) RNA-seq library preparation and RNA-sequencing, 2) Characterization of module topology, 3) Cell counting to estimate RiboTag expression fidelity, 4) Modification of the DIAMOnD method, 5) Validation of the DIAMOnD method, 6) Cell counting to estimate RiboTag expression fidelity, 7) Immunohistochemistry, 8) Immunofluorescence. (DOCX) [file ppat.1010747.s001.docx]

Supplementary methods

# Immunohistochemistry

Formalin fixed brain tissues were treated with 98% formic acid to reduce prion infectivity, and then post-fixed for at least four more days in formalin. Brains were then embedded in paraffin with each cassette containing both NBH and RML injected brains, ensuring that controls and diseased samples were stained identically. The necessity of formic acid pretreatment renders the brain samples in a fragile state that is most successfully prepared with paraffin sectioning. These methods result in high autofluorescence and reduce the usefulness of the samples for immunofluorescent studies. We therefore employed chromogenic staining methods for all scrapie-infected and control samples. Cassettes were cut into 4µm thick sections. Sections were dewaxed in xylene and rehydrated in graded dilutions of ethanol (each 5min). Sections stained for PrP aggregates were further treated with 98% formic acid (3 min) rinsed with water twice, neutralized with PBS RT, 5 min, digested with 10mg/ml proteinase K for 30 min, 37C, then washed three times with PBS. This step was excluded for all other stainings. Epitope retrieval was performed with a steamer in 0.01 M citrate buffer (PrP: pH = 6; Iba1, GFAP: pH = 8; 30min). Endogenous peroxidase was removed with

H2O2 treatment (3%, 5 min). For PrP and GFAP staining Mouse on Mouse Elite Peroxidase Kit (Vector Laboratories) was used according to the manufacturer’s instructions. For Iba1 staining 2,5% donkey normal serum (S30, Merck Millipore) was used for blocking (30min), followed by incubation with the primary antibody (3060min), biotinylated secondary antibody (30min) and AB-Complex (30 min). Staining of the epitope was done with DAB with Nickel for all stainings (DAB Peroxidase (HRP) Substrate Kit with Nickel, Vector Laboratories; 5-10 min). A counterstain with hematoxylin (H-3404, Vector Laboratories) was performed (10 s) and sections were dehydrated with graded dilutions of ethanol and xylene (each 5 min). For each time point, all brain sections were treated at the same time with the same solutions and materials. Pictures of sections were made with an AxioCam camera mounted onto a Zeiss AXIO Observer.A1 microscope with Zen 2012 software used with the same microscope imaging parameters for related stainings and magnifications. Primary antibodies: Anti-GFAP Clone GA5 (MAB360), 1:5000, Merck Millipore; PrP^res^ (Prion Protein) monoclonal antibody SAF84, 1:200, Cayman Chemical; Rabbit Anti Iba1 for ICC, 1:200, Waco; Rabbit anti Rps21, 1:100, Bethyl (A305-070A); secondary antibodies: Biotin-SP-conjugated AffiniPure Donkey Anti-Rabbit IgG, 1:500, Jackson Immuno Research.

# Immunofluorescence

In order to assess the cell type-specificity of RiboTag experiments, we processed additional mice for immunohistological evaluation. Mice were perfused transcardially with saline followed by 10% neutral buffered formalin solution (Sigma) and immersionpostfixed in the same solution overnight at 4C. After cryoprotection in 30% sucrose in PBS, 40 µm coronal cryosections were taken and sections stored until use in 50% 0.1 M PBS, 30% ethylene glycol, and 20% glycerol at -20C. Sections at the level of AP-1.46 (Paxinos and Franklin, 2nd edition) were selected, and double incubated overnight at 4°C in primary antibodies against HA (1:200, 3F10, Sigma) and the respective marker antibody (Somatostatin, 1:200, HPA019472, Atlas; Sox9, 1:500, AB5535, Millipore; NeuN, 1:500, MAB377, Millipore; Parvalbumin, 1:200, Novus, NB120-11427; Satb2, 1:200, Abcam, 92446; GFAP, 1:500, Dako, Z0334; Iba1, 1:100, Wako, 019-19741; Gad67, 1:2000, Acris, AP15571PU). The double incubation for HA and Gad67 was preceded by a 45 min antigene retrieval in pH6 citrate buffer using a vegetable steamer (15 min + 30 min cooling to RT), quenching of peroxidases in 0.3% H2O2, and avidin-biotin block (SP-2001, Vector). Visualization occurred by double incubation in fluorescent secondary antibodies from Jackson Immun Research against rat IgG (712-165-153 or 712-545-153) and appropriate second antibody (mouse, 715545-151; rabbit, 711-225-152 or 711-585-152). For Gad67 labeling we instead used a biotinylated secondary antibody (711-065-152) followed by tyramide signal amplification (T20935, Thermo Fisher).

# Cell counting to estimate RiboTag expression fidelity

To assess the overlap between cells expressing the HA-tag and cells immunopositive for select markers, we counted cell profiles in hippocampus and cortex. A picture of 320x320 µm was taken with a confocal microscope (LSM 700, Zeiss) using a 20x objective and all labelled cell profiles counted. For the hippocampus, we selected CA1. In cases where CA1 showed no or very few labelled cells, we analyzed the hilus region. For the cortex, we selected the deep layers right above CA1. If the number of HA+ cells was low, an additional second or third picture was taken, so the minimal number of HA+ cells in one analysis was 10. For images selected for publication, brightness and contrast were enhanced using ImageJ.

# RNA-seq library preparation and RNA-sequencing

300ng of total RNA or 150ng of immunoprecipitated mRNA was used for RNAsequencing. Each sample was checked for amount and quality using a Nanodrop 2000 (Thermo Fisher Scientific) and Agilent 2100 Bioanalyzer. For each condition (5 Cre/Rtag lines, injected with RML or NBH, sacrificed at 10 or 18 WPI) we used four individual samples (exceptions: SST RML 10 WPI: n = 3; PV NBH 10 WPI: n = 3; Astro RML 18 WPI: n = 2). RNA was converted to cDNA using the Transcriptor High Fidelity cDNA Synthesis Kit (Roche Aplied Science). RNA-sequencing libraries were prepared using the TruSeq RNA Sample Preparation 2 Kit (Illumina). The library quality was checked using an Agilent 2100 Bioanalyzer and concentration was measured by a Qubit dsDNA HS Assay Kit (Thermo Fisher Scientific) and adjusted to 2nM before sequencing (single end, 50 bp) on a HiSeq 2000 Sequencer (Illumina) using TruSeq SR Cluster Kit 3-cBot-HS (Illumina) and TruSeq SBS Kit 3-HS (Illumina) according to the manufacturer’s instructions.

# Modification of the DIAMOnD method

First, we constructed a PPI network using the STRING database (87). We

considered all interactions in the filtered networks, regardless of the species in which they were primarily described. Although we didn’t choose to further trim the interactome data, we observed that only a fraction of seed genes has corresponding vertices in the PPI network which may serve as proof of its incompleteness (Table S2). However, we found that most of the seeds occupy neighboring interactome positions (Table S2). With consideration of the shortcomings of the PPI networks, we sought to develop an algorithm relying on the connectivity patterns of seed genes as well as on the information contained in gene expression data. We chose to modify the existing DIAMOnD algorithm (32). For every candidate gene, we considered the significance of the change in its expression levels (Wald test p-value from DEseq2) and connectivity significance as a measure of the gene’s topological relevance. Specifically, we have identified all seed gene neighbors and ranked them according to their respective connectivity significance p-values and differential expression (Wald test) p-values. The individual rankings were then combined into a single integrated score. The gene with the highest score was included in the growing disease module. As in the original algorithm, each identified gene was considered a member of the seed cluster in the following iteration (see also “Construction and validation of cell type-specific disease modules” in the main methods). This approach prevents omissions of potential genes of interest that could simply be excluded due to the following possibilities: 1) conservative criteria selection of DEGs based solely on an arbitrary significance threshold, 2) changes to gene expression being too subtle to be detected due to low statistical power inherent in settings with a large number of variables and a relatively low number of biological replicates, 3) subthreshold changes of expression of several genes belonging to a functional set, but having a significant cumulative effect on the network 4) changes to certain genes not being reflected by

RNA levels (e.g., protein stability affected instead) or 5) changes involving a different regulatory modality (e.g., phosphorylation). All algorithms used are available on GitHub (https://tinyurl.com/5sbexp89).

# Validation of the DIAMOnD method

We integrated the interactome and RiboTag data sets using a modification of the DIAMOnD method (32) and sought to identify putative disease modules specific to each cell type in early pre-symptomatic disease stage (10 WPI). To compare the performance of the default and the modified DIAMOnD version, we first specified a second, less stringent arbitrary significance threshold (Wald test p-value from DEseq2 < 0.01) to define a set of genes that are likely to be differentially expressed but do not meet the seed cluster inclusion criteria and consider them as part of a module validation cluster (putatively regulated genes). The rationale for this approach was that if DEGs are to be primarily found at the top of a gene list ranked according to differential expression p-values, a disease module should be enriched in genes from the top of the p-value ranking. Figure S5C shows that more putatively regulated genes were found by the modified diamond method in 500 iterations. Moreover, the rate at which new putatively regulated genes were added to the module was higher up to a threshold (Fig 5C) that was used as the iteration cutoff for formulating the final modules.

# Characterization of module topology

Topological characteristics of generated graphs for the original and modified DIAMOnD methods were determined with the following functions of the igraph R package: gorder() (number of nodes), gsize() (number of edges), components (number and size of connected components), diameter() (size of the longest of all shortest paths in the module), mean_distance() (average length of all pairwise shortest distances), edge_density() (the number of edges divided by the number of possible edges). The genes constituting individual disease modules constructed with the modified DIAMOnD algorithm were assigned to unique communities using the fast greedy modularity optimization algorithm (cluster_fast_greedy() function in igraph package). Each module gene set with size ≥10 was then tested for enrichment with GO terms. The term with the lowest Benjamini Hochberg adjusted p-value was chosen as a representative term of each community. Additional results of the GO analysis of Cx43, vGluT2 and Gad2 modules are presented in Figs S6, S7 and S8 (respectively). A comparison of results using the original and modified DIAMOnD methods is presented in Fig S9.
