# Supplementary figures and images for: Distinct translatome changes in specific neural populations precede electroencephalographic changes in prion-infected mice
Source: PLoS Pathog. 2022 Aug 12;18(8):e1010747. doi: 10.1371/journal.ppat.1010747 (PMC9401167; doi:10.1371/journal.ppat.1010747)

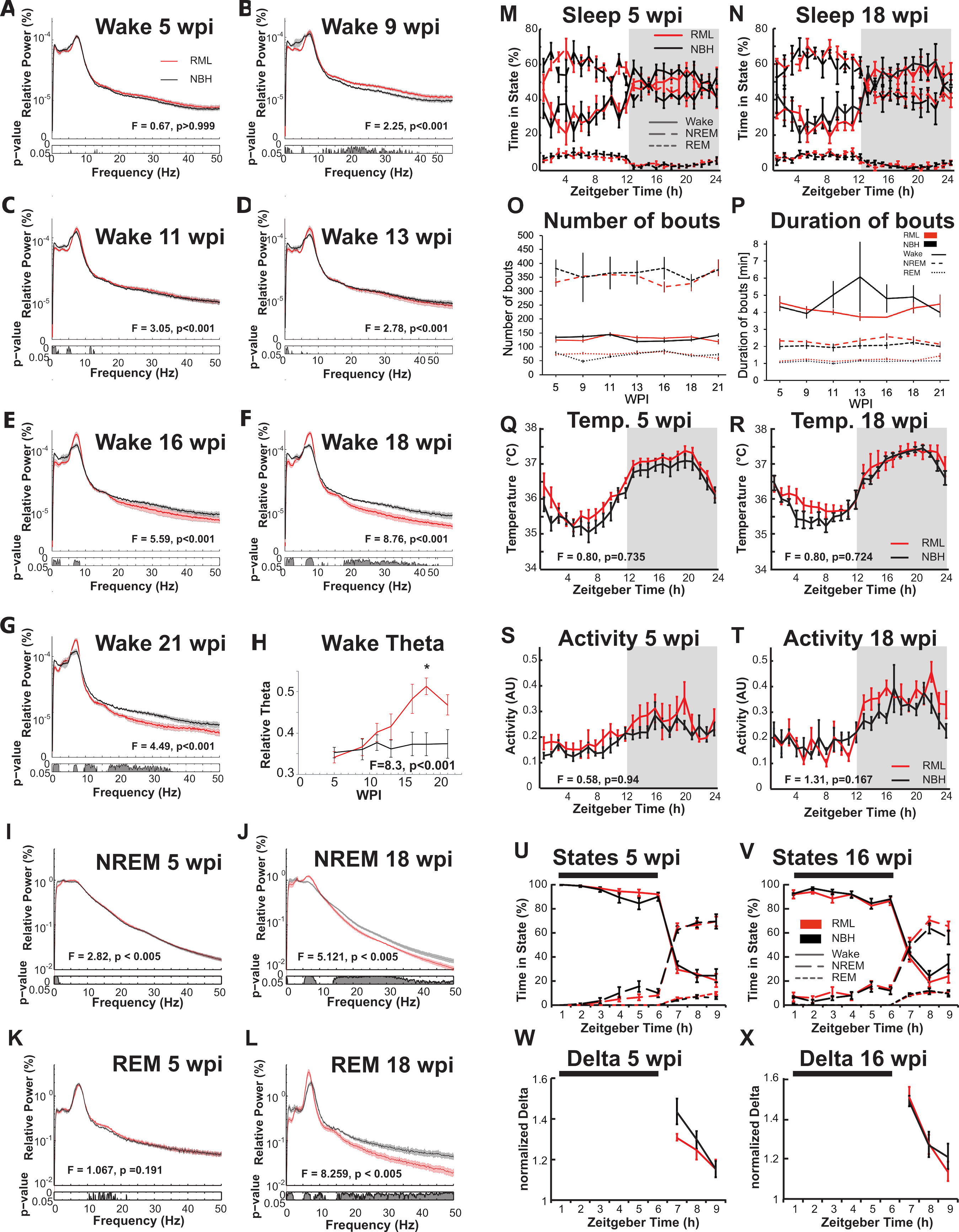

Supplement: S1 Fig — (A-L) Frequency power spectra from serial EEG. (A-G) Examples of frequency power spectra during wake, acquired serially as disease progressed. The power in each frequency bin is expressed as percentage of the cumulative power of all frequencies (0–50 Hz). Curves depict group averages, shaded areas depict SEM. The degrees of freedom are 409 and 3272. The p-values for post hoc uncorrected bin-by-bin t-tests are indicated below the spectra. The average wake power spectra up to 11 WPI (C) shows no difference between the groups but at 13 WPI (D) differences begin to emerge and at 18 WPI (F) RML-infected mice show a significant increase in relative power in the theta range (5–10 Hz) and a decrease in relative power in lower and higher frequencies. A summary of data in panels A-G is depicted in H, also shown in Fig 1C. A similar increase in theta at 18 WPI was also seen for NREM (J) and REM (L), but not at 5 WPI (I, K). (M-T) Baseline vigilance states, bout properties, temperature and activity in RML and control mice, relative to time. (M, N) Average Wake, NREM and REM time during 24 h undisturbed recording. The shaded area indicates the 12 h lights-off period. Mixed model ANOVA did not reveal significant main effects for group (RML or NBH) nor interactions between group and Zeitgeber Time (ZT, hourly bins) for any vigilance state at either 5 WPI (M) or 18 WPI (N). (O-P) Longitudinal analysis of mean bout number (O) and mean bout duration (P) of REM (dotted line), NREM (dashed line) and wakefulness (solid line) bouts across all timepoints. Error bars depict SEM. No significant differences were found. (Q, R) Average intraperitoneal temperature across 24 h undisturbed recording. The average temperature did not differ between groups (no main effect found by ANOVA), and the course of temperature over time was also not affected by treatment (no interaction of temperature and ZT) at 5 and 18 WPI (Q and R, respectively). The ANOVA result for the interaction is given at the bottom [file ppat.1010747.s002.tiff]

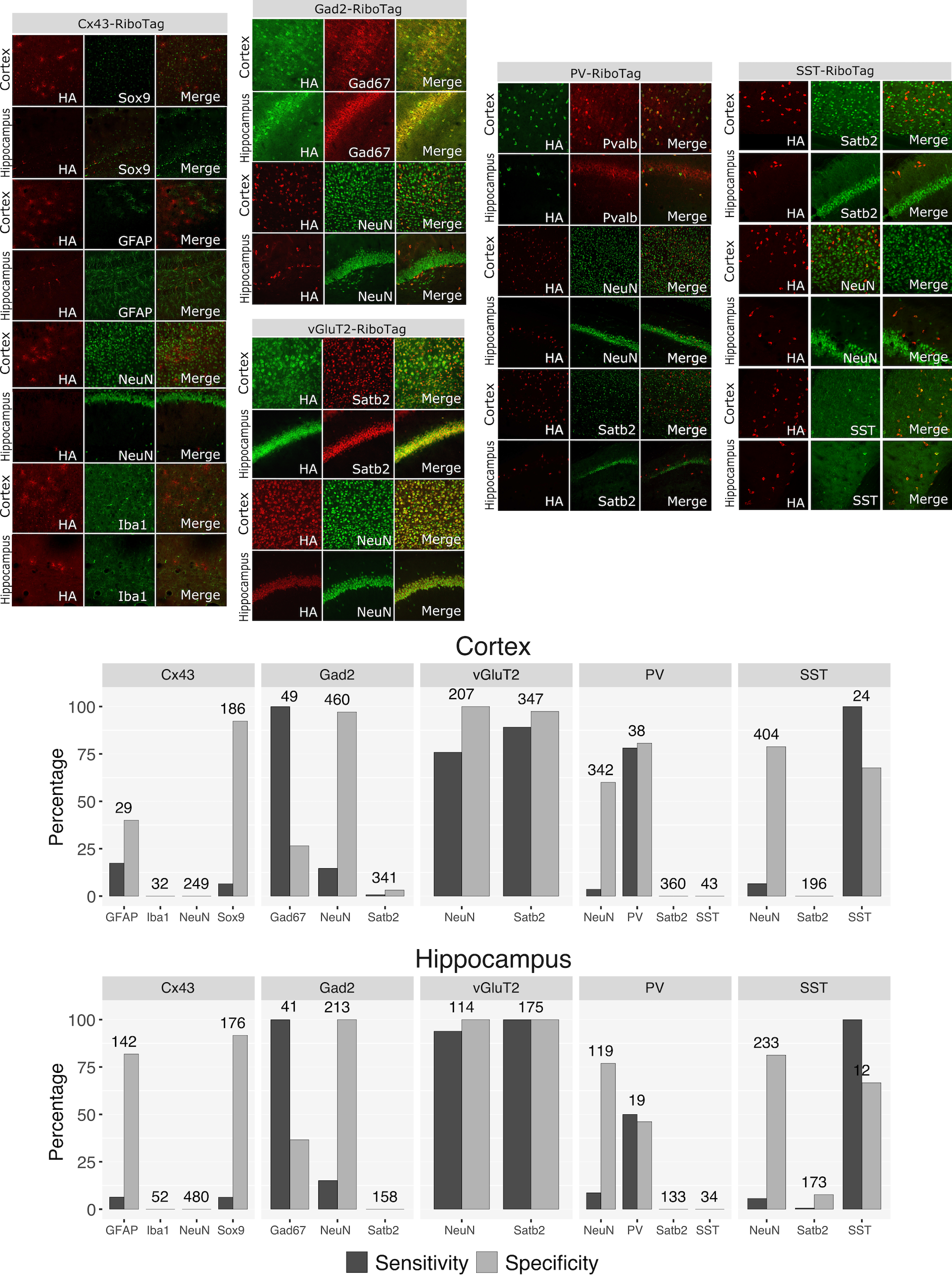

Supplement: S2 Fig — (A) Confocal images of double immunofluorescence labeling of HA (RiboTag) and representative histological cell-type markers. (B) Bar plots showing the numbers of cells immunopositive for HA and appropriate histochemical markers. Sensitivity is the percentage of cell profiles positive for the marker that is also positive for HA. Specificity is the percentage of cell profiles positive for HA that is also positive for the marker. (TIFF) [file ppat.1010747.s003.tiff]

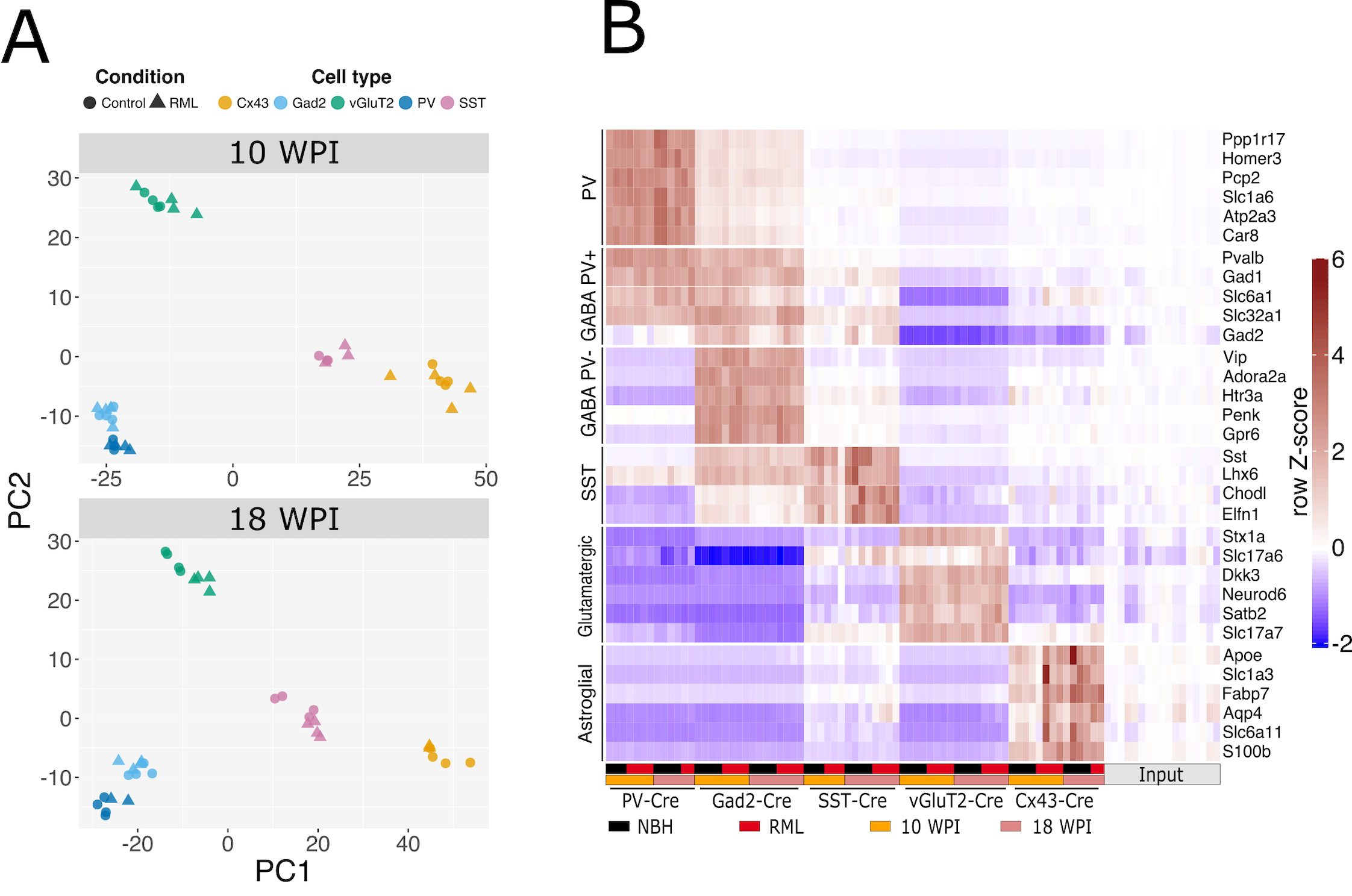

Supplement: S3 Fig — (A) Principal Component Analysis (PCA) of RiboTag data from all cell types and time points analyzed. PCA was done on variance-stabilized normalized counts (vst() transformation using DEseq2 R package). Clustering was dominated by differences between cell types rather than disease states. (B) Heatmap showing RiboTag specificity. Each row corresponds to a cell type marker for one of the cell types listed on the left side of the heatmap. Each column represents one biological replicate (one mouse), and the specification of the sample is encoded by the colored legend below the heatmap. Input samples are total RNA collected specifically for this analysis for comparative purposes. Z-score for each row was calculated so that the mean input level for each row was set to 0 (Z = (x–row_meaninput)/row_SD, where row_SD is row standard deviation and x is the TPM value). (TIFF) [file ppat.1010747.s004.tiff]

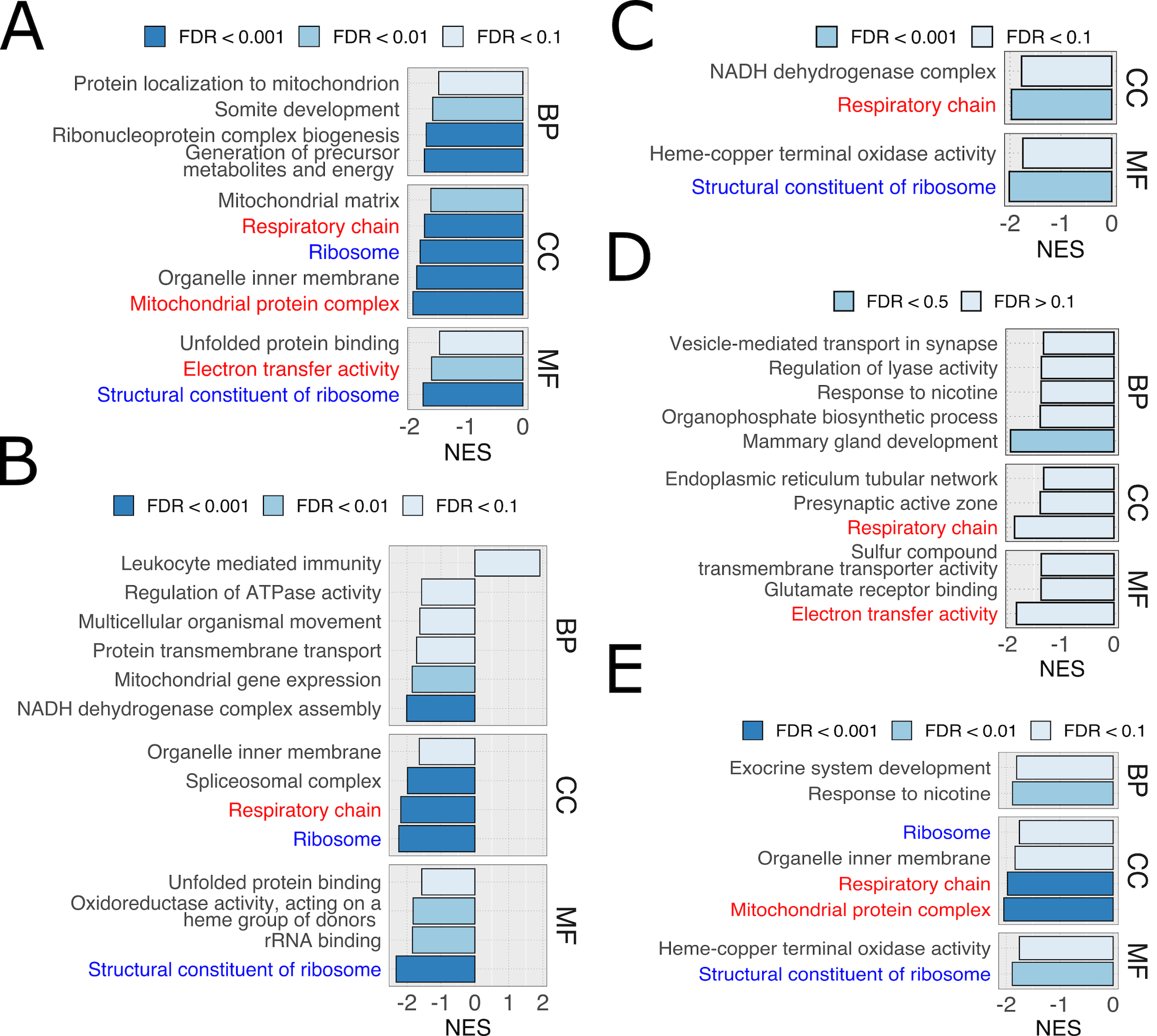

Supplement: S4 Fig — (A-E) GSEA of DEGs in (respectively) astrocytes, glutamatergic neurons, SST neurons, PV neurons and GABAergic neurons at 18 WPI. In case of PV neurons (D), no terms enriched at FDR < 0.1 were found and therefore all reported terms were shown. Categories with GSEA FDR < 0.1 were shown after removing similar terms based on semantic similarity using GOSemSim R package. Categories related to terminal oxidation (highlighted in red) and translation (highlighted in blue) were amongst the most enriched, and were common across multiple analyzed cell types, suggesting that the 18 WPI timepoint is marked by canonical changes to mitochondria and ribosomes, typical for neurodegenerative diseases. (TIFF) [file ppat.1010747.s005.tiff]

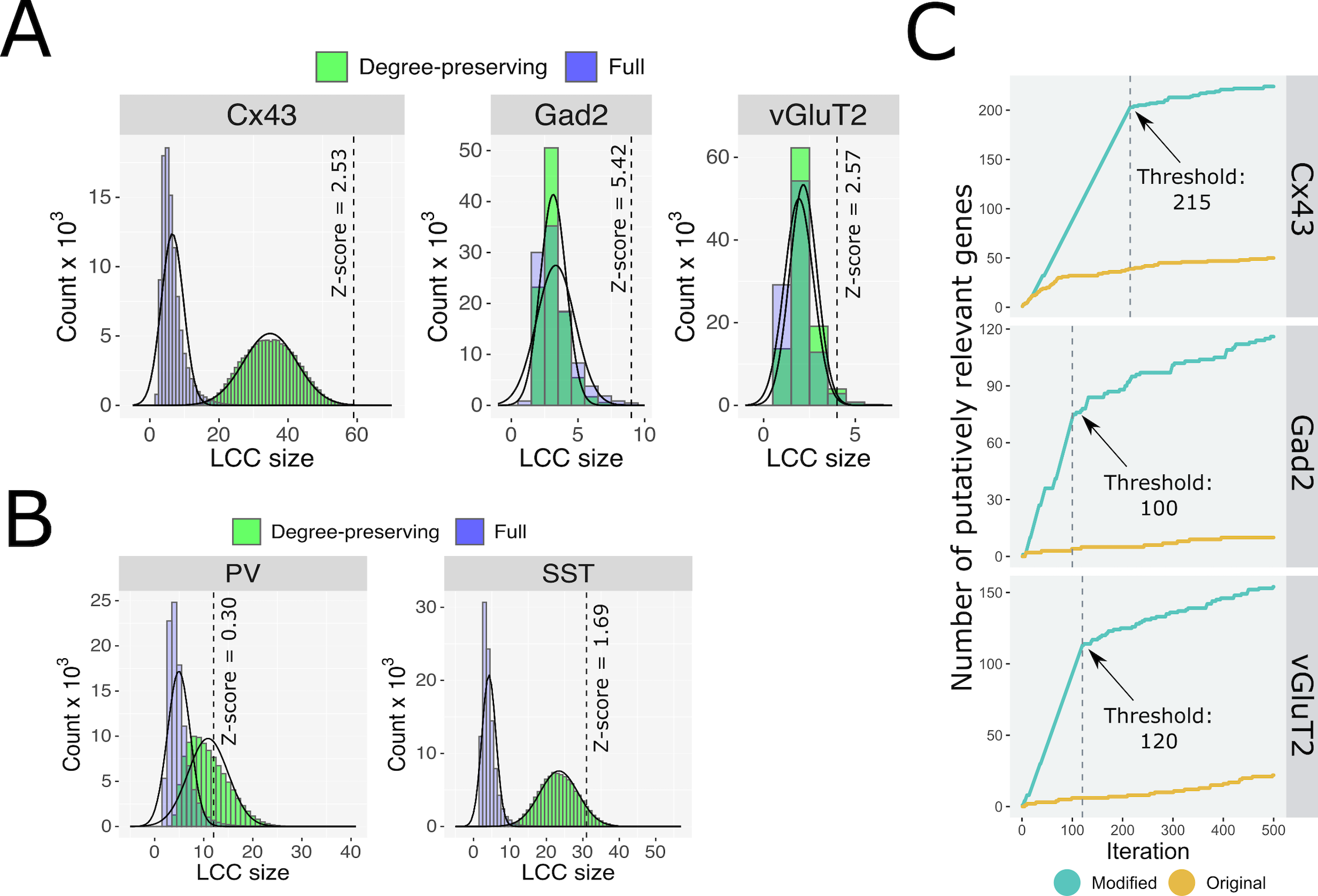

Supplement: S5 Fig — (A, B) LCC sizes of randomly generated networks in which random genes either preserved the degrees from of the original network (degree-preserving randomization) or not (full randomization). Vertical dotted lines correspond to the LCC size of the original network with Z-scores with respect to the degree preserving distribution (green histograms). (A) For affected cells (Cx43, Gad2 and vGluT2), LCC sizes were larger (indicated by dotted lines) than control (random) LCCs computed using either method. (B) For PV and SST cells, LCCs that were not larger than the degree-preserving controls. (C) Determination of lists sizes was based on the breakpoint threshold resulting from repeated additions of one gene at each iteration. (TIFF) [file ppat.1010747.s006.tiff]

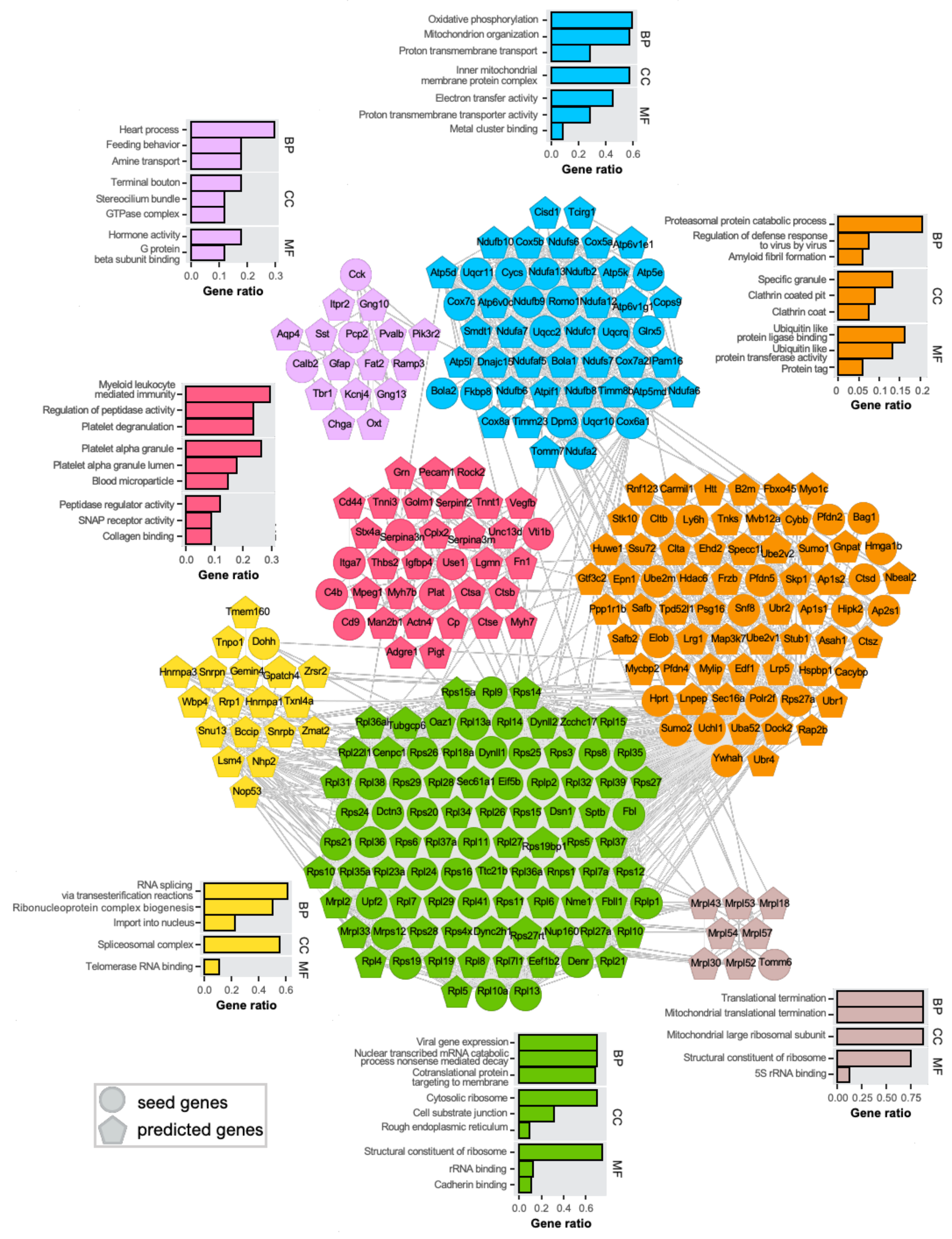

Supplement: S6 Fig — Colors represent fast-greedy clustering of genes within the module. (TIFF) [file ppat.1010747.s007.tiff]

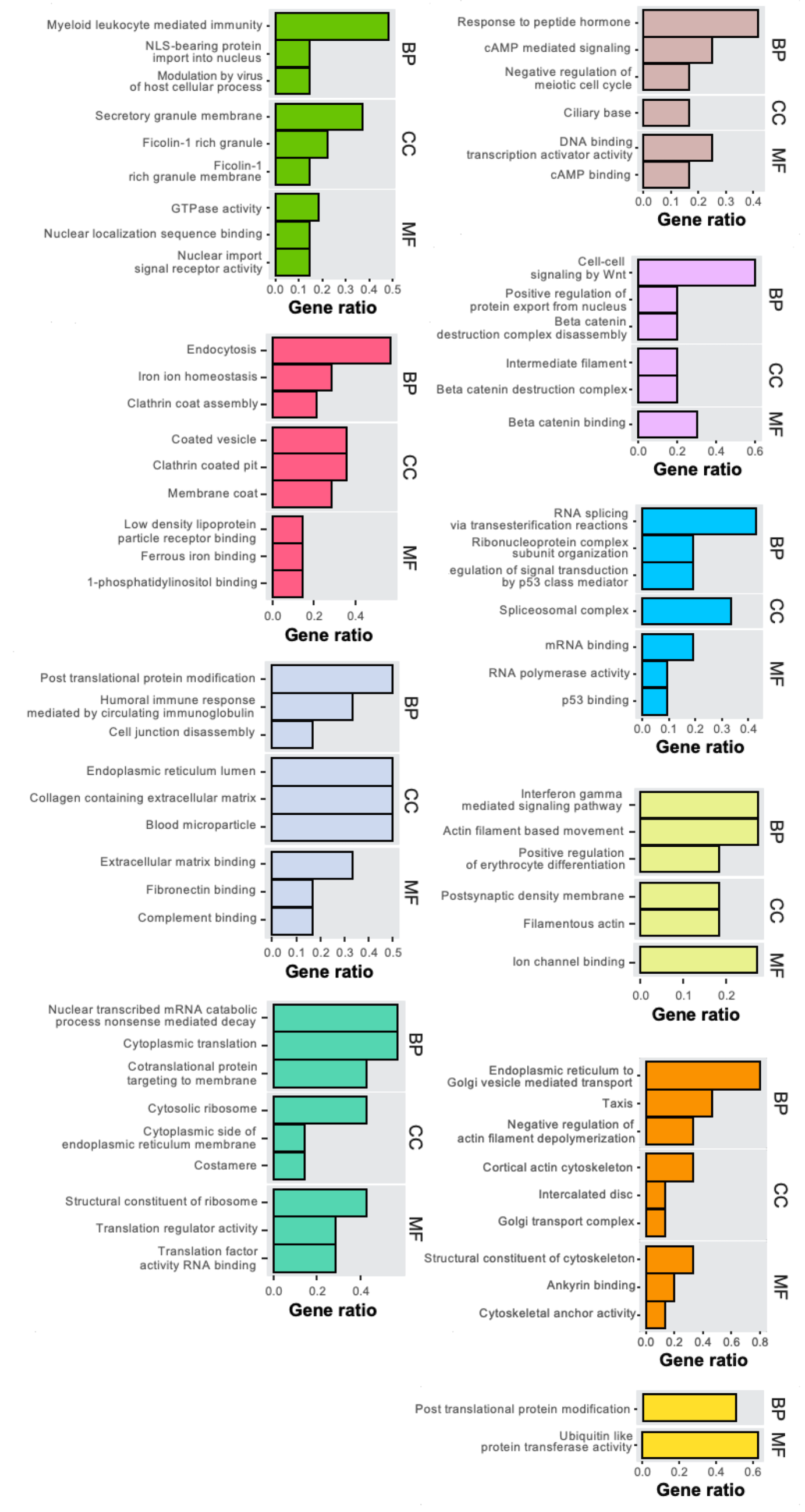

Supplement: S7 Fig — Colors represent fast-greedy clustering of genes within the module. (TIFF) [file ppat.1010747.s008.tiff]

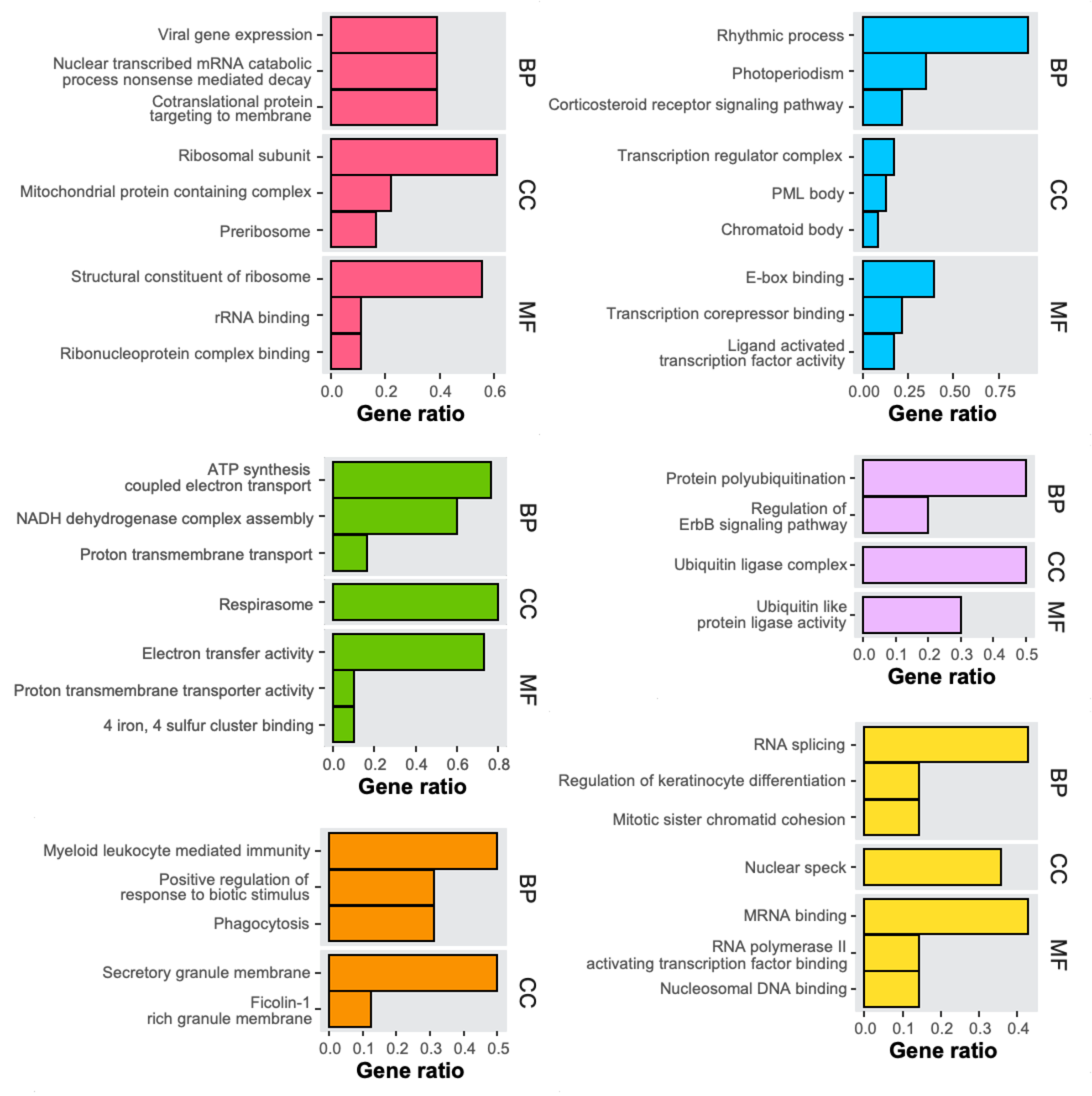

Supplement: S8 Fig — Colors represent fast-greedy clustering of genes within the module. (TIFF) [file ppat.1010747.s009.tiff]

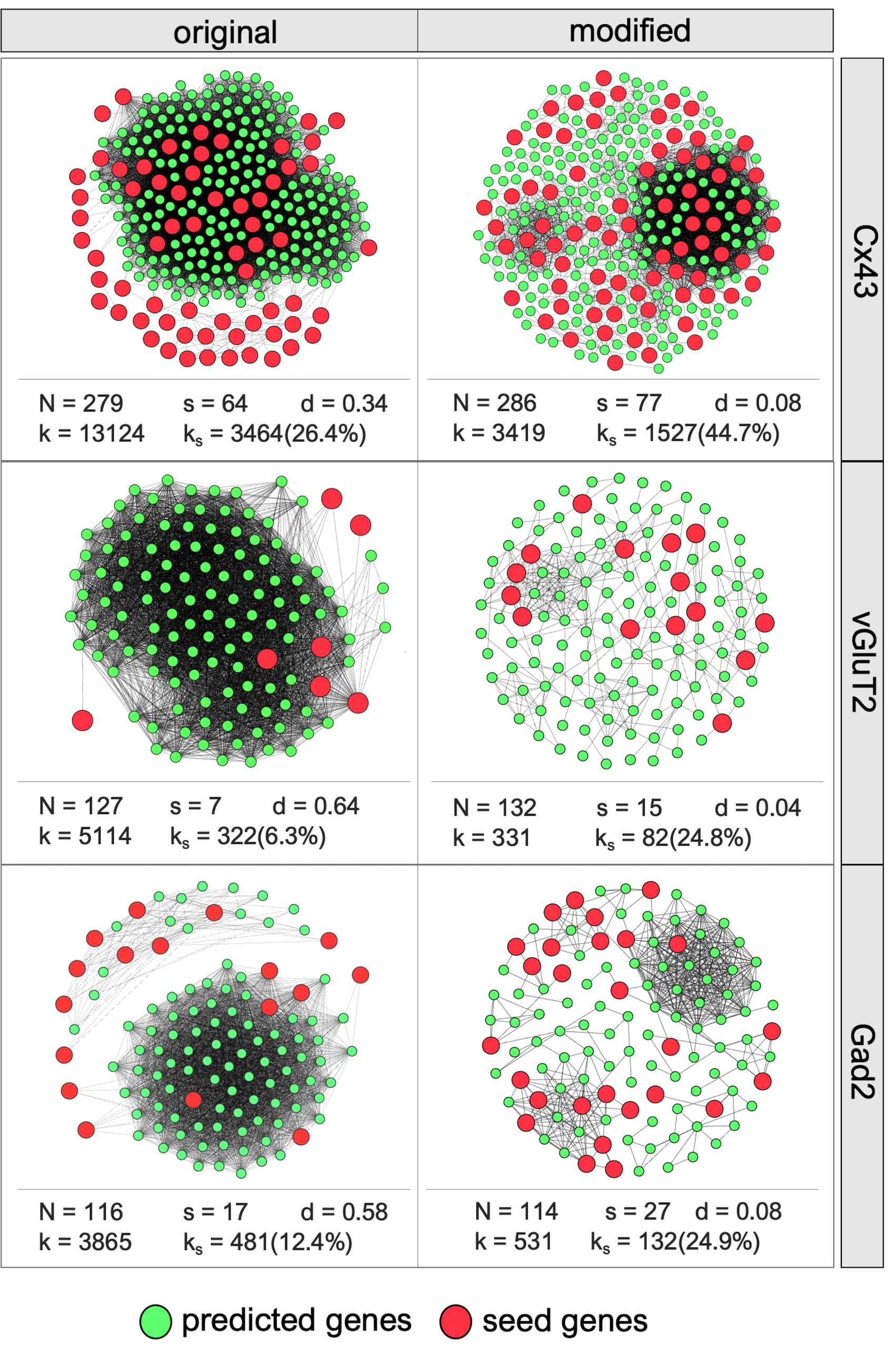

Supplement: S9 Fig — Topological characteristics of disease modules obtained with original (left) and modified (right) DIAMOnD algorithm. N—number of nodes, s—number of seed nodes in the final module, d—edge density, k—number of edges, ks—number of edges including predicted and seed nodes (ks). The modules produced by the modified algorithm had more seed genes and a higher percentage of connections between initial seed genes and predicted genes than those obtained from the original algorithm. (TIFF) [file ppat.1010747.s010.tiff]
