# Supplementary material for: Distinct translatome changes in specific neural populations precede electroencephalographic changes in prion-infected mice
Source: PLoS Pathog. 2022 Aug 12;18(8):e1010747. doi: 10.1371/journal.ppat.1010747 (PMC9401167; doi:10.1371/journal.ppat.1010747)
Supplement: S1 Table — In contrast to a previous report [90], we observed that node removal does not have a major impact on network connectivity. As indicated by the number of connected components, filtered networks are more fragmented in comparison to the generic network. However, most of the genes constitute a single connected component (96.9% on average). The diameter and mean shortest distance did not increase with node removal, suggesting that the “small world” property of the original network was preserved. (PDF) [file ppat.1010747.s011.pdf]

Table S1. Topological properties of PPI networks.

	Number of nodes	Number of edges	Size of LCC	Number of connected components	LCC diameter	LCC mean distance
**Original PPI network**	16181	364969	15767	152	15	3.955
**Cx43**	12800	267375	12401	271	13	3.862
**Gad2**	12636	264921	12248	273	13	3.852
**vGluT2**	12557	259357	12160	286	13	3.866
