# Supplementary material for: Distinct translatome changes in specific neural populations precede electroencephalographic changes in prion-infected mice
Source: PLoS Pathog. 2022 Aug 12;18(8):e1010747. doi: 10.1371/journal.ppat.1010747 (PMC9401167; doi:10.1371/journal.ppat.1010747)
Supplement: S2 Table — Only a fraction of seed genes have corresponding vertices in the PPI network. Most of the seeds occupy neighboring interactome positions, as indicated by the significance of their LCC size (empirical p-value < 0.05). Note that sizes of LCCs formed by seeds were compared to a reference distribution which was obtained by measuring the LCC size of random genes with degrees similar to the ones of the initial seed cluster. Thus, the observed local clustering cannot be attributed solely to the network’s structural characteristics, but points to the network localization of the seed genes themselves, highlighting their relevance in marking the disease-related interactome community. (PDF) [file ppat.1010747.s012.pdf]

Table S2. Seed gene information and validation results.

	Number of seed genes	Number of seed genes in the PPI network	Observed LCC size	Empirical p-value
**Cx43**	137	128	55	0.0048
**Gad2**	83	69	9	0.0014
**vGluT2**	38	33	4	0.0426
